# Editorial: What's next? Innovative translational markers across the ALS-FTD continuum

**DOI:** 10.3389/fnins.2023.1250127

**Published:** 2023-08-14

**Authors:** Fabiola De Marchi, Pilar Maria Ferraro, Alessandro Introna, Edoardo Gioele Spinelli

**Affiliations:** ^1^Department of Neurology and ALS Centre, University of Piemonte Orientale, “Maggiore della Carità Hospital”, Novara, Italy; ^2^Neurology Unit, IRCCS Ospedale Policlinico San Martino, Genova, Italy; ^3^Department of Translational Biomedicine and Neuroscience “DiBraiN”, University of Bari “Aldo Moro”, Bari, Italy; ^4^Neuroimaging Research Unit, Division of Neuroscience, IRCCS San Raffaele Scientific Institute, Milan, Italy; ^5^Department of Neurology, Vita-Salute San Raffaele University, Milan, Italy

**Keywords:** amyotrophic lateral sclerosis, frontotemporal dementia, biomarkers, precision medicine, risk factor

Amyotrophic Lateral Sclerosis (ALS) and Frontotemporal Dementia (FTD) are considered part of a common clinical and neuropathological continuum, with the hallmarks of both diseases being present within the same person in the so-called ALS-frontotemporal spectrum disorder (ALS-FTSD). Over the last decades, a growing amount of research has led to a better characterization of the ALS-FTSD, considering the common genetic, molecular, neuroradiological and neuropathological signatures and the significant inter-individual heterogeneity in clinical presentation and evolution. So, the potential of available markers for ALS diagnosis and prognostic estimation continues to improve. However, to date, no specific markers for the definition or prediction of motor disability or cognitive/behavioral dysfunction have been validated. For example, the current Strong criteria define ALS-FTSD from a clinical and neuropsychological standpoint but will soon need the support of wet biomarkers (both on CSF and plasma) and translational approaches provided by neuroimaging and neurophysiology at different levels of complexity. For this reason, many research efforts in this area are dedicated to the search for translational markers able to define this neurodegenerative spectrum better.

In this Frontiers Research Topic, the four published articles will help the researchers to be updated on the main innovations in this area, speeding up the applicability of these findings. In this Volume, most studies focused mainly on ALS, where the underlying pathological mechanisms are even more obscure, but translatable to the whole spectrum.

Starting from the diseases' risk, we know that in FTD a positive family history for dementia is reported in roughly 40% of cases, although a clear autosomal dominant transmission accounts for only 10% of cases. On the contrary, non-genetic risk factors are yet to be identified, although traumatic brain injury, bipolar disorder, diabetes mellitus and thyroid diseases are associated with an increased risk of disease (Bang et al., 2015).

Instead, the acquired risk factors for ALS are more investigated. As widely accepted, only a small percentage of cases have a clear Mendelian transmission. A more likely combination of genetic and acquired factors is responsible in most cases. The comprehensive meta-analysis by Duan et al. also delved into the interplay between genetic and non-genetic factors for the risk of developing ALS. Using a systematic review methodology for study inclusion, which considered both European and Asian populations, their results confirmed the role of well-known risk factors such as pesticide exposure, heavy metals, and solvents (OR > 1.3). Subgroup analysis also clarified that current smoking significantly increased the risk of ALS, compared with previous smoking, suggesting that lifestyle modifications might impact the individual risk of disease development. Moreover, the significant protective associations that were found with high BMI and the use of antidiabetic drugs strongly support the potential importance of metabolic and nutritional interventions for a protective effect on ALS, in line with previous suggestions (Dupuis et al., 2011; Vasta et al., 2021). Subgroup analyses also clarified the heterogeneity of genetic risk factors in sporadic and familial forms of ALS and between European and Asian populations, highlighting, in any case, the importance of a thorough genetic screening to stratify risk in unaffected family members.

Besides risk factors, research focuses on previously unexplored pathological mechanisms possibly involved in disease onset and evolution. In this context, the review by Castelnovo et al. summarized current evidence on the role of basal ganglia (BG) alterations in the clinical evolution of ALS, highlighting the need further to investigate brain structures traditionally unexplored in the disease to get a deeper understanding of its underlying pathophysiology. In particular, the up-to-date overview of structural and functional Magnetic Resonance Imaging studies in the field provided converging evidence from different imaging modalities on the association between BG degeneration and motor and cognitive symptoms of the disease.

Recent important updates are not confined to risk factors and newly discovered pathological processes but include disabling symptoms relatively underestimated in ALS and new reworkings of the classical electromyographic diagnostic process, possibly leading to novel neurophysiological markers.

Pain is a frequently underrated ALS symptom that has been largely investigated in recent works (Chiò et al., 2017), considering its potential impact on quality of life. An et al. extensively discussed this topic in their paper. First, the authors confirmed the high frequency of pain in this large cohort of Chinese ALS patients. Surprisingly, the pain did not affect the quality of life, assessed with an innovatively multidimensional scale evaluating different—often ignored—domains. Conversely, anxiety, depression, and spinal burden can affect the quality of life in ALS patients. The social beliefs and ethics of the country of origin could have affected the impact of pain on the quality of life, resulting from complex and heterogeneous mechanisms (Ciećwierska et al., 2023). Anyway, the latter findings highlight the importance of paying attention to limb dysfunction to detect and treat pain in an adequate and timely manner. Connected to this point, and always regarding the clinical setting, Zoccolella et al. described current evidence regarding the split-hand and split-leg phenomena in a concise and easily consultable mini-review, focusing on the possible associated neurophysiological findings and their clinical implications. The authors' findings supported the role of these signs as reliable markers for motor neuron disease differentiation and prognosis estimation. In fact, although the exact underlying mechanisms related to the split indexes are yet unclear, the authors support the idea on an involvement of both cortical and spinal pathways for their genesis.

In conclusion, the present Frontier Research Topic focused on research updates in ALS-FTSD, ranging from risk factors to imaging and clinical disease markers, consolidating concepts already well-known in the literature, and hypothesizing new underlying paths to investigate. All these reported detecting points will help improve diagnosis, staging, and prognosis, heading toward a path to precision medicine that needs to be implemented soon.

## Author contributions

FD conceived and wrote the manuscript with great scientific input from PF, AI, and ES. All authors contributed to the article and approved the submitted version.
